# Anxiety, Depression, and Obsessive-compulsive Disorder in a Recently Diagnosed Case of Systemic Sclerosis

**DOI:** 10.7759/cureus.4748

**Published:** 2019-05-24

**Authors:** Omair A Khan, Ramsha Majeed, Kaynat Kamal, Mahnoor Sherazi, Muhammad Saad

**Affiliations:** 1 Internal Medicine, Fauji Foundation Hospital, Rawalpindi, PAK; 2 Public Health, Health Services Academy, Quaid-E-Azam University, Islamabad, PAK

**Keywords:** anxiety, systemic sclerosis, obsessive compulsive disorder, depression

## Abstract

Systemic sclerosis is an autoimmune condition that frequently affects women. It is a progressive, debilitating disease that has widespread manifestations, targeting different organs of the body with potentially fatal consequences due to lung and kidney involvement. Women with this disease mostly present with Raynaud’s phenomenon along with symptoms of gastro-esophageal reflux disease (GERD). Just like any chronic debilitating condition, patients with systemic sclerosis often suffer from mental health issues that can further worsen their condition, significantly affecting their quality of life. Further research regarding the effects and severity of the disease should be encouraged for a better understanding of the illness, its diagnosis, and treatment. We present a rare case of a 55-year-old woman who presented with complaints of a major depressive episode. She was diagnosed with systemic sclerosis last year and has a history of generalized anxiety disorder. She was prescribed Mirtazapine, an antidepressant. On her follow-up after one month, she started complaining of obsessive ruminations that were causing her significant distress. She was prescribed an add-on therapy with a selective serotonin reuptake inhibitor (SSRI) antidepressant with the emphasis being placed on cognitive behavioral therapy (CBT). She reported an improvement in her obsessive symptoms as well as depression after two months.

## Introduction

Systemic sclerosis, an autoimmune disorder, is characterized by the buildup of fibrosis under the skin and in internal organs [1]. Fibrosis is caused by increased formation of collagen, a protein that supports and strengthens connective tissues throughout the human body. This increased fibrosis starts to reside in internal organs leading to either impairment of the affected organ or complete failure [2]. Commonly affected organs are esophagus, lungs, heart, and kidneys [3]. The onset of symptoms usually occurs between the age group of 25 and 55 years. Systemic sclerosis presents with Raynaud’s phenomenon preceding fibrosis. The affected individual’s fingers and toes start to turn white or blue, as a result of impairment in small vessels carrying blood to these areas, in response to external temperature stimuli [4]. This is followed by swelling and inflammation of hands and feet, ultimately leading to the thickening of the overlying skin due to fibrosis. People with systemic sclerosis may or may not have ulcers on their fingers and painful swollen pimples under their skin [5].

The prevalence of systemic sclerosis worldwide is reported to range from 50 to 300 cases per one million people [6]. Although the risk of developing sclerosis exists for all males, females, children, and elderly people alike, women are four times more prone to developing the disease. This can further be supported by the fact that in pregnant women, the risk is increased by ten-fold [7].

Systemic sclerosis is mostly characterized to be sporadic, meaning that it occurs in people with no family history of the said condition. Yet there are reported cases of other autoimmune disorders in close relatives of the affected individuals. A small percentage of cases of systemic sclerosis are familial with no clear inheritance pattern [8].

Skin afflictions, particularly systemic sclerosis, result in body changes that contribute to the development of psychological impairments such as anxiety, depression and obsessive-compulsive disorder. Patients of systemic sclerosis usually exhibit symptoms of psychological distress, characterized by changed physical appearance, fatigue, severe pain, and difficulty in daily occupations, ultimately affecting the quality of life of the patient [9]. Living with a painful illness, tiredness, and constant fatigue in the face of daily activities and changed physical appearance form a firm bridge for the formation of feelings such as demoralization, low self-esteem, loss of self-worth, hopelessness and suicidal thoughts. Studies have confirmed increased psychiatric symptoms in patients with systemic sclerosis, but painful symptoms associated with systemic sclerosis are insufficient to explain the high prevalence of psychiatric comorbidity in patients with systemic sclerosis [10]. According to one study, the cerebral environment of systemic sclerosis was similar to that of schizophrenic patients, hence proposing a directly proportional relationship between systemic sclerosis and psychiatric distress [11]. However, further studies covering ethnicity and severity of both the diseases as well as environmental factors are needed for better understanding as well as the development of better diagnostic and prognostic measures.

## Case presentation

A 55-year-old woman presented to the OPD with complaints of anxiety, insomnia, fatigue, decreased appetite, anhedonia, lack of concentration, lack of energy, and low mood. She did not report any suicidal intentions. Her Becks Depression Index (BDI) score was calculated to be 32, which comes under the category of severe depression. She gave a history of generalized anxiety disorder which was diagnosed 17 years ago, for which she was prescribed an SSRI and a benzodiazepine for a few years until her condition stabilized. She was diagnosed with systemic sclerosis a year back and has been in constant distress because of it. She was taking bosentan twice daily to control her blood pressure and Raynaud’s phenomenon. She was also prescribed a disease-modifying anti-rheumatic drug (DMARD) called hydroxychloroquine to control associated joint problems. She did not report any symptoms of mania or hypomania, though she claims to have lived all her life with occasional anxiety. Due to her symptoms, she has lately been having a hard time doing regular house chores and has stopped teaching at the local school where she works as a teacher. There is no history of substance abuse or any psychiatric conditions in the patient’s family. After careful review, she was diagnosed with a major depressive disorder and was started on a first line atypical antidepressant mirtazapine, which also allowed the patient to sleep better. She was also prescribed a benzodiazepine to counter any associated anxiety and insomnia until the mirtazapine took effect. During her next follow-up after one month of treatment, the patient continued complaining about depressive symptoms along with intrusive thoughts which had started one week earlier. These intrusive thoughts were related to her faith, occurring many times during the day and causing her significant distress and making her question her faith. These intrusive thoughts were making her anxious as well, leading to impairment in her everyday life. She did not report any compulsions with these obsessive ruminations and hence she was diagnosed with pure obsessional obsessive-compulsive disorder. Her score on the Yale-Brown Obsessive-Compulsive Scale (Y-BOCS) was 14, which comes under the category of mild OCD. Due to this, an SSRI escitalopram was added to her treatment regimen for her associated obsessive ruminations, as SSRIs are considered the first-line drugs for OCD. She was also advised to undergo cognitive behavioral therapy (CBT) which has proven to be beneficial in the treatment of obsessive-compulsive disorder (OCD). Due to the limited CBT practitioners in the area, she did not undergo CBT and relied solely on pharmacotherapy. At her regular follow-up, she showed improvement and was advised to continue treatment. 

## Discussion

Systemic sclerosis or scleroderma is an autoimmune disorder that affects the skin and internal organs. It is characterized by an accumulation of fibrous tissue in the skin and other organs, due to the protein collagen. The most prominent sign includes episodes of Raynaud’s phenomenon, in which the fingers and toes of an individual turn white or blue due to exposure to stress or cold temperatures. Other signs and symptoms of the disease include swollen hands, sclerodactyly, telangiectasia, open ulcers, and calcinosis. Fibrosis can also affect internal organs, most commonly affecting the heart, lungs, kidneys, and esophagus. Symptoms indicating internal organ damage include dysphagia, heartburn, shortness of breath, hypertension, diarrhea or kidney problems [12]. Diagnosis is based on the presence of antinuclear antibodies particularly anti-centromere and anti scl-7o antibodies. There is no cure for systemic sclerosis, only symptomatic management can be offered. Drugs used in the management of systemic sclerosis include methotrexate, corticosteroids, and NSAIDs.

Although the subject is one that lacks adequate research, previous studies have indicated that patients with systemic sclerosis often experience various psychiatric comorbidities. One study conducted in 2001 revealed the occurrence of a myriad of psychiatric conditions in the majority of a group of 33 female patients who were suffering from systemic sclerosis. These psychiatric conditions included depression, anxiety, obsessive-compulsiveness, as well as somatization and feelings of guilt [13]. Another study conducted in France between 2002 and 2004 was done on a group of 100 women who were known cases of systemic sclerosis. It was found that they too showed a high rate of psychiatric symptoms in association with systemic sclerosis. Depression and anxiety disorders were the main culprits, with 19% of the patients complaining of ongoing episodes of depression at that time. It was observed that 56% experienced a major depressive episode at some point in their life, with 14% admitted to experiencing dysthymia. Out of the 100 women, anxiety disorders were diagnosed in 37. Despite this high rate, only half of these women were given appropriate treatment for their psychiatric symptoms [14].

*Depression *in patients of systemic sclerosis is a debilitating condition that is strongly linked to personality and extent of emotional support provided [15]. It should be promptly diagnosed and treated. According to DSM-5, an individual must exhibit five or more of these symptoms in a period of two weeks [16]:

· Depressed mood most of the day, nearly every day.

· Markedly diminished interest or pleasure in all, or almost all, activities most of the day, nearly every day.

· Significant weight loss when not dieting or weight gain, or decrease or increase in appetite nearly every day.

· A slowing down of thought and a reduction of physical movement (observable by others, not merely subjective feelings of restlessness or being slowed down).

· Fatigue or loss of energy nearly every day.

· Feelings of worthlessness or excessive or inappropriate guilt nearly every day.

· Diminished ability to think or concentrate, or indecisiveness, nearly every day.

· Recurrent thoughts of death, recurrent suicidal ideation without a specific plan, or a suicide attempt or a specific plan for committing suicide.

OCD is a distinctive form of anxiety characterized by distressing thoughts and repetitive behaviors or rituals. The DSM-5 diagnostic criterion for OCD entails the following [17]:

A. Presence of obsessions, compulsions, or both:

*Obsessions *are defined as:

 · Recurrent and persistent thoughts, urges, or images that are experienced, at some time during the disturbance, as intrusive and unwanted, and that in most individuals cause marked anxiety or distress.

 · The individual attempts to ignore or suppress such thoughts, urges, or images, or to neutralize them with some other thought or action (i.e., by performing a compulsion).

*Compulsions *are defined as:

 · Repetitive behaviors (e.g., hand washing, ordering, checking) or mental acts (e.g., praying, counting, repeating words silently) that the individual feels driven to perform in response to an obsession or according to rules that must be applied rigidly.

 · The behaviors or mental acts are aimed at preventing or reducing anxiety or distress, or preventing some dreaded event or situation; however, these behaviors or mental acts are not connected in a realistic way with what they are designed to neutralize or prevent, or are clearly excessive.

B. The obsessions or compulsions are time-consuming (e.g., take more than one hour per day) or cause clinically significant distress or impairment in social, occupational, or other important areas of functioning.

C. The obsessive-compulsive symptoms are not attributable to the physiological effects of a substance (e.g., a drug of abuse, a medication) or another medical condition.

D. The disturbance is not better explained by the symptoms of another mental disorder (e.g., excessive worries, as in generalized anxiety disorder; preoccupation with appearance)

*Generalized anxiety disorder (GAD)* is a condition that is often difficult to diagnose. The DSM-5 indicates the criteria for helping in its diagnosis. When assessing for GAD, clinical professionals are looking for the following [18]:

 I. The presence of excessive anxiety and worry about a variety of topics, events, or activities. Worry occurs more often than not for at least 6 months and is clearly excessive.

 II. The worry is experienced as very challenging to control. The worry in both adults and children may easily shift from one topic to another.

 III. The anxiety and worry are accompanied with at least three of the following physical or cognitive symptoms (In children, only one symptom is necessary for a diagnosis of GAD):

· Edginess or restlessness

· Tiring easily; more fatigued than usual

· Impaired concentration or feeling as though the mind goes blank

· Irritability (which may or may not be observable to others)

· Increased muscle aches or soreness

· Difficulty sleeping (due to trouble falling asleep or staying asleep, restlessness at night, or unsatisfying sleep)

The prompt diagnosis and management of these psychiatric conditions are necessary to help improve the quality of life of patients. For the treatment of depression, medications and psychotherapies are the best options [19]. Self-help techniques such as exercising, socializing, sleeping better, etc., are also beneficial in the case of females. Among the best treatments offered for OCD patients, the leading pharmacotherapy remains to be SSRIs, while the best psychotherapy is CBT administered by a trained professional. The treatment for GAD is a combination of counseling and medications such as antidepressants, benzodiazepines, azapirones, anti-convulsants, and anti-psychotics. Our patient currently suffers from two out of the three psychiatric conditions mentioned, that is, depression and OCD, and has a past history of the third which is GAD. She was started on 15 mg mirtazapine initially, which was then increased to a dose of 45 mg which improved her major depressive episode. She did experience a few documented side effects of mirtazapine such as dry mouth and sedation. In fact, mirtazapine was purposely selected for this patient due to its ability to sedate the patient and help with insomnia. For her OCD, an SSRI escitalopram was given at a low dose of 10 mg, which proved to be effective in controlling her symptoms within a month. Benzodiazepine was also given in the meantime to help her anxiety, which proved to be effective. The combined sedative effect of mirtazapine and benzodiazepine helped the patient to combat insomnia and sleep a minimum of 8-9 hours a day. 

## Conclusions

Systemic sclerosis, like any other chronic autoimmune condition, not only affects a person physically but also has psychological and emotional consequences which can hinder a person’s ability to lead a healthy life. Psychiatric screening must be considered in patients diagnosed with systemic sclerosis. Healthcare professionals are encouraged to be more vigilant in recognizing the early signs of mental health problems like depression, anxiety and OCD, which once established are further aggravated by chronic autoimmune diseases as illustrated by this case. Timely diagnosis and management will not only drastically improve the quality of life for these patients, but will also play a key role in the prevention of debilitating sequelae.
